# The Effect of Dopaminergic Medication on Impulse Control and Compulsive Behaviour: A Translational Perspective

**DOI:** 10.1111/bcpt.70264

**Published:** 2026-06-14

**Authors:** Mirjam Wolfschlag, Elena Espa, Kevin Oliveira Hauer, Jonathan Timpka, Ruben Smith, Per Odin, Maria Angela Cenci, Anders Håkansson

**Affiliations:** ^1^ Clinical Addiction Research Unit, Psychiatry, Department of Clinical Sciences Lund, Faculty of Medicine Lund University Lund Sweden; ^2^ Region Skåne, Malmö Addiction Center Skåne University Hospital Malmö Sweden; ^3^ Basal Ganglia Pathophysiology Unit, Department of Experimental Medical Science, Faculty of Medicine Lund University Lund Sweden; ^4^ Clinical Memory Research Unit, Department of Clinical Sciences Malmö, Faculty of Medicine Lund University Lund Sweden; ^5^ Region Skåne, Memory Clinic Skåne University Hospital Malmö Sweden; ^6^ Division of Neurology, Department of Clinical Sciences Lund, Faculty of Medicine Lund University Lund Sweden; ^7^ Region Skåne, Department of Neurology Skåne University Hospital Lund Sweden

**Keywords:** dopamine agonist, impulse control disorder, impulsive‐compulsive behaviour, Parkinson's disease, restless legs syndrome

## Abstract

Dopaminergic medication used in disorders like Parkinson's disease (PD) and restless legs syndrome can cause impulsive‐compulsive behaviour (ICB), often with strong negative effects on patients' quality of life. This narrative review presents translational evidence on iatrogenic ICB, taking findings from epidemiological, clinical, neuroimaging and preclinical studies into consideration. Epidemiological and clinical studies find dopamine agonists with high D2/3‐selectivity to be most strongly linked to ICB. Their effect on ICB has often been shown to be dose‐dependent, but the impact of combining different dopaminergic drugs or applying extended‐release formulations is less clear. Intervention studies support tapering or replacing dopamine agonists for ICB reduction, whereas no efficacious pharmacotherapy has been identified for ICB treatment specifically. Adequate animal models for mimicking different types of ICB are available, and point, in line with human neuroimaging studies, towards an involvement of striatum and prefrontal cortex in iatrogenic ICB. Overall, complementary research designs have led to profound evidence regarding the occurrence of ICB in PD and establishing methods transferable to other, less‐studied patient populations. A combined approach integrating insights from human studies and animal models could contribute to developing dopaminergic drugs with lower ICB risk but also specific pharmacotherapies for impulsivity or compulsivity in the future.

## Plain Language Summary

Diseases like Parkinson's disease and restless legs syndrome are treated with drugs that affect dopamine activity in the brain. As a side effect, these drugs can lead to a lower impulse control, manifested, for example, as gambling disorder or hypersexuality. This article summarises research on these side effects, collected through a variety of scientific methods. The drug type with the highest risk for behavioural side effects has been identified, but many details remain unclear, especially in patients with other disorders than Parkinson's disease. Results from both human brain imaging and animal models start to reveal brain pathways involved.

## 1 Introduction

Dopaminergic medication is widely used in neurological, psychiatric and endocrine conditions [1]. In Parkinson's disease (PD), motor symptoms caused by the loss of dopaminergic neurons are treated with dopamine replacement therapy [2, 3]. Classically, this therapy consists of the dopamine precursor levodopa and/or dopamine receptor agonists like pramipexole, ropinirole and rotigotine. In addition, other dopaminergic drug types including monoamine oxidase‐B (MAO‐B) or catechol‐O‐methyltransferase (COMT) enzyme inhibitors and amantadine can be applied. Moreover, dopaminergic drugs, especially pramipexole, are also used in restless legs syndrome (RLS) and fibromyalgia [4, 5], and the dopamine agonist cabergoline is a treatment common in pituitary disorders [6]. For psychiatric patients, partial dopamine agonists like aripiprazole have been developed as antipsychotics, mainly prescribed in schizophrenia and bipolar disorder [7].

Treatment with dopaminergic agents can lead to impulsive‐compulsive behaviour (ICB) as an adverse event [1, 2, 8]. ICBs include impulse control disorders like pathological gambling, compulsive sexual behaviour, compulsive shopping and overeating, but also stereotypic behavioural patterns like hoarding, punding and hobbyism [2]. Furthermore, patients can develop an addiction‐like condition in response to their dopaminergic medication, called dopamine dysregulation syndrome. ICBs often have a detrimental impact on the patient's mental health but can even severely compromise physical health, personal relationships, and their financial situation [2, 9]. Thus, ICBs as adverse events of dopaminergic medication tend to contribute substantially to the disease burden.

Iatrogenic ICB has been explored from many angles and through a variety of methods. Existing evidence has been reviewed with a clinical [2] or preclinical perspective [10], respectively, and also within the field of neuroimaging studies [11]. However, an overarching, translational summary is missing to date. The objective of this narrative review was therefore to present an overview of ICB developing under dopaminergic pharmacotherapy, not including deep brain stimulation in PD, based on knowledge stemming from different types of study designs.

For this narrative review, the main original studies within each field were selected to be presented in the respective chapter after a thorough, recent review of the literature by the expert authors for each methodological approach; see, for example, [12]. Priority was given to the key studies contributing with the most substantial evidence for advancing their field. In included human studies, the outcome ICB was determined by patients' self‐reports or clinical diagnostic assessments, such as diagnosis codes or the Questionnaire for Impulsive‐Compulsive Disorders in Parkinson's Disease (QUIP). In animal models, ICB was assessed by behavioural tests mimicking specific psychological features of ICB. More specifically, search terms were aimed at the exposure dopaminergic medication and the outcome ICB, and included terms like ‘dopaminergic treatment’, ‘dopaminergic therapy’ or ‘dopamine agonist’ in combination with ‘impulse control disorder’ or ‘compulsive behaviour’.

Articles considered for the clinical chapters were screened for each methodological subtype, which resulted in 16 epidemiological/pharmacovigilance studies, 293 observational studies and 21 interventional studies. Criteria for their selection were based on the strength of evidence (size of study population and study design) and the most relevant findings in line with the scope of this review. Regarding animal studies, 121 articles were screened, and the final selection was made on the following hierarchical approach: We filtered for studies utilising validated PD models (6‐hydroxydopamine or adeno‐associated viral vector overexpression of alpha‐synuclein), in presence of chronic dopaminergic regimens with pramipexole, ropinirole or another agonist coadministered with levodopa, and using behavioural paradigms with high translational value and/or mechanistic insights. For the neuroimaging chapter, the terms ‘fMRI’, ‘[15O]H2O PET’, ‘[11C]raclopride’, ‘[18F]fallypride’, ‘[18F]DOPA’, ‘PET’ or ‘SPECT’ were added to the search strategy and 180 articles were screened. The final selection of the studies described in this section was based on the authors' assessment of the studies judged most relevant to the scope of this review.

The following chapters constitute a summary of studies with an epidemiological, clinical (observational and interventional), preclinical and neuroimaging approach. While presenting the main contributions of each design, an additional goal was to identify strengths and limitations of each method for studying iatrogenic ICB. We hope to provide a guide to open research questions and at the same time to which methods might be most suitable to target them in the future.

## 2 Epidemiology and Pharmacovigilance

To gain an understanding for ICBs under dopaminergic treatment on a population‐wide level, health care registers and pharmacovigilance reporting systems constitute valuable sources of information. By accessing precollected, systematic databases, they offer the opportunity to examine much larger study populations than feasible in clinical studies. In some cases, not only cross‐sectional but also longitudinal or cohort‐like approaches are possible [1, 13]. One benefit of screening reports of ICBs as adverse events is that the patient population is typically not limited to one indication for dopaminergic treatment but all underlying diagnoses are included [14, 15].

A main disadvantage of epidemiological studies on iatrogenic ICB is that they can only provide a large‐scale overview but rarely individual information like specific symptom development or dopaminergic doses prescribed. Few patients with ICB symptoms receive a formal ICB diagnosis, making it harder to detect these types of problems in health care registers and often leading to a considerable underestimation of ICB prevalences [9]. In addition, pharmacovigilance databases rely on the active initiative to report adverse events by patients, relatives or health care staff. It can therefore be assumed that many cases of iatrogenic ICB stay undetected, for example, due to the patient not recognising the ICB as an adverse effect of their dopaminergic medication.

The major contribution of pharmacovigilance and register studies to the field of iatrogenic ICB has been to systematically confirm the clinical observation of ICB presenting under dopaminergic treatment on a population‐wide level. Moreover, they have helped to detect the drugs most strongly linked to ICB and to understand which diagnostic groups might be at highest risk for iatrogenic ICB.

In pharmacovigilance and register studies, especially dopamine agonists are associated with the occurrence of ICB as adverse event, both the full agonists used in PD, RLS, pituitary disorders and fibromyalgia, but also the partial agonists used as antipsychotics [1, 5, 13]. Across all indications, dopamine agonists with high affinity for D2/3 receptors, like pramipexole, ropinirole or aripiprazole, are related to ICB as adverse event more often [1, 15, 16]. One study even proposed a direct, positive correlation between reports of impulse control disorders and the levels of D3 receptor occupancy by full dopamine agonists, as inferred from pharmacokinetic public online databases [17].

Most pharmacovigilance reports identify PD patients as the major patient group affected by ICB occurring as adverse event of dopaminergic therapy, in contrast to RLS patients constituting the largest patient group exposed to dopaminergic treatment. One explanation could be the lower dopaminergic doses used in RLS, possibly reducing the ICB risk. However, as mentioned above, these results might be biased by an underreporting of ICB as adverse event in non‐PD populations, in which ICB is much less recognised and monitored than in PD patients. According to Moore et al. [1], in 2014, 62% of all reports of dopamine agonist‐related ICB occurred in PD patients, only 24% in RLS and 4% in conditions related to hyperprolactinemia. Similar proportions of underlying indications for dopaminergic medication were later confirmed by Fusaroli et al. [17]. The same research group found that patients with mood disorders were more often affected by ICB under the treatment with aripiprazole than patients with psychotic disorders [14]. In comparison, and although one must keep in mind that ICB has been studied much more thoroughly in PD than in other diseases, ICB prevalences have been found to be similar across different indications for dopaminergic medication by observational clinical studies. Prevalence estimates commonly range between 15% and 35% in PD patients [2, 3, 12] and have been found to lie around 7%–40% in RLS populations [4, 12, 18]. In pituitary disorders, the ICB prevalence has been reported between 7% and 61% [6, 12] and for psychotic disorders, no systematic assessment of ICB prevalence is available to date.

## 3 Observational Clinical Studies

Observational clinical studies constitute the most commonly applied design for studying iatrogenic ICB and have thus created the major body of evidence in the field. They have led to a comprehensive understanding of ICB in PD during the last 25 years [2], enabling evidence‐based guidelines for ICB management [19]. Furthermore, observational studies have provided some information on ICB during dopaminergic therapy in other study populations such as patients with RLS, pituitary and psychotic disorders [4, 6, 7].

A cross‐sectional design provides the possibility to study ICB prevalences in large populations, analysing different ICBs, exposures to dopaminergic medication or patient characteristics [3, 4, 20]. In addition, the international comparison between different national populations and ethnicities has mainly been enabled by cross‐sectional studies. However, cross‐sectional studies lack a temporal perspective and provide associations rather than causal relationships regarding factors that influence the development of ICB. Cohort and other prospective studies, on the other hand, take longitudinal changes into consideration and have contributed considerably to understanding the ICB risk of specific dopaminergic treatment strategies [21, 22, 23]. As a third observational study design, case–control studies have been widely used to investigate iatrogenic ICB. Being able to control for confounders like sex, age at PD onset or psychiatric comorbidity, they are well suited to identify an ICB risk factor profile specifically associated with dopaminergic treatment [7, 24]. Nevertheless, similar to cross‐sectional designs, case–control studies can rarely investigate the causality between risk factors and the development of ICB.

### 3.1 High‐Risk Dopaminergic Agents for Developing Impulsive‐Compulsive Behaviour

As indicated by epidemiological studies, dopamine agonist treatment has been associated with ICB across all patient populations under dopaminergic therapy [3, 4, 6, 7, 25]. Many studies point out D2/3 receptor agonists to carry an especially high risk for ICB [20, 21, 26], and the D2/3 agonists pramipexole and ropinirole are in general considered to be equally strong risk factors for ICB [3, 21, 23]. In comparison, levodopa therapy has widely been shown to represent a safer option in the context of ICB [3, 21, 23, 25, 27]. Adding a dopamine agonist to levodopa therapy, a common clinical practice in PD, has been found to increase the ICB risk in some studies [3, 22, 27, 28]. However, other studies did not confirm that combined levodopa and dopamine agonist therapy was an ICB risk factor [21, 25]. Dopaminergic drug classes other than dopamine agonists and levodopa have been investigated less well regarding their effect on ICB. Most studies attribute a low ICB risk to MAO‐B inhibitors [23, 25, 28], but there are some contradictory findings [26]. Amantadine, a compound acting on multiple receptor targets, has been found to increase the ICB risk in most cases [28, 29].

### 3.2 Effect of Dopaminergic Dose, Treatment Duration and Pharmacokinetics

Multiple studies have investigated a dose effect of dopaminergic treatment on iatrogenic ICB with mixed results. Still, most evidence points towards an increased ICB risk with increasing dopamine agonist doses in PD patients [3, 21]. Sharma et al. [28] showed an association between ICB occurrence as adverse event and higher dopamine agonist doses. This association was found for both pramipexole and ropinirole individually, whereas Staubo et al. [24] showed an association with higher doses and serum concentrations of ropinirole but not of pramipexole. A Spanish cohort study found a dopamine agonist dose effect on the risk for developing ICB, including a correlation between higher dopamine agonist doses and higher ICB rating scores [23]. However, not all studies detect an impact of the dopamine agonist dose on ICB in PD [25]. In RLS patients, one study indicates a similar association of higher dopamine agonist doses with an increased ICB risk as in PD patients [4]. In contrast, two studies in patients with pituitary or psychotic disorders, respectively, found no dopamine agonist dose effect on ICB rates [6, 7].

Compared with the acknowledged effect of dopamine agonist doses, an overall dose effect of the dopaminergic treatment has been more controversial. Some studies find an increased ICB risk with increased total dopaminergic doses [28], whereas others show no effect on ICB occurrence [23, 25]. In addition, findings on the impact of dopaminergic treatment duration vary. Sharma et al. [28] found an association between an increased ICB risk and longer treatment duration with levodopa, dopamine agonists in general and pramipexole and ropinirole specifically. Other studies confirmed a higher ICB risk under longer dopamine agonist treatment [21, 24], yet there are many negative findings regarding an ICB effect of dopaminergic treatment duration as well [6, 7, 23]. Another aspect that has been proposed to influence the risk for developing ICB under dopaminergic medication have been extended‐release in comparison to immediate‐release formulations for dopamine agonists. To date, some evidence for a protective effect of extended‐release formulations of pramipexole exists [20], but other studies show an equal ICB risk increase independent of the type of dopamine agonist formulation [26, 27].

## 4 Clinical Intervention Studies

Interventional designs are much less commonly applied to study iatrogenic ICB than observational approaches and have, to our knowledge, only been performed in PD populations so far. They provide the highest evidence level but are often limited by small sample sizes with isolated findings that are not repeated systematically in larger populations. International guidelines recently published by an expert consortium have summarised recommendations for the management of ICB during PD therapy, partly based on evidence from intervention studies [19]. For mild to severe impulse control disorders, dopamine agonist tapering or discontinuation is suggested as the first line approach. If unsuccessful, even other dopaminergic agents like levodopa or MAO‐B inhibitors should be reduced in dose. In a third step, cognitive behavioural therapy, advanced therapies like deep brain stimulation, which enable a reduction in dopaminergic treatment, or the use of antipsychotics or antidepressants can be considered. For punding and dopamine dysregulation syndrome, a reduction in levodopa treatment is suggested as the primary option.

### 4.1 Adaptations to Dopaminergic Therapy

Given the nature of studying ICB as an adverse event of a drug class, some interventions have focused on changing the dopaminergic medication. In line with findings from non‐interventional designs across different patient populations [21], the reduction or cessation of dopamine agonist treatment have shown efficacy in improving iatrogenic ICB. Lee et al. [30] showed that an intervention of substituting dopamine agonist treatment with levodopa treatment for 12 weeks reduced impulsivity scores in PD patients with ICB. This improvement was strongest in patients experiencing compulsive sexual behaviour, but several ICB patients showed an unexpected increase in impulsivity scores instead. Another strategy by Lyons et al. [31] has been to add the MAO‐B inhibitor selegiline to the dopaminergic treatment, allowing a reduction in dopamine agonist use. This led to a complete resolution of ICB in a third of all patients and a reduction in an additional 50%. Despite a broad consensus that dopamine agonist tapering is effective in managing iatrogenic ICB, only few intervention studies have targeted this research question. Little is known about the efficacy of specific changes to dopaminergic treatment, such as dose–response effects or the combination of specific drugs.

### 4.2 Additional Pharmacotherapy for Managing Impulsive‐Compulsive Behaviour Under Dopaminergic Treatment

Intervention studies beyond adapting dopaminergic therapy for ICB management have focused on a few non‐pharmacological approaches like psychotherapy, transcranial stimulation or multidisciplinary rehabilitation, but mainly on additional pharmacotherapy with other drug classes. One randomised controlled trial in PD patients showed that clonidine, an α2‐adrenergic receptor agonist with vasodilating, sedative and analgetic effects, but not placebo treatment reduced severity ratings of ICB after 4 and 8 weeks [32]. However, numbers of patients in full remission were comparable in both treatment arms. Another randomised controlled trial in PD patients found a larger decrease in ICB severity ratings under treatment with the opioid antagonist naltrexone, a drug used to manage alcohol and opioid use disorders, than under placebo treatment [33]. An exploratory study reported that, adding the centrally acting psychostimulant atomoxetine to dopaminergic treatment reduced impulsivity and risk taking in PD patients with ICB [34]. Another small, exploratory study in PD patients found that adding the treatment with the antiepileptic drug zonisamide to PD medication reduced ICB severity and general impulsiveness [35]. In summary, only single studies in small PD patient populations have investigated additional pharmacotherapy as an option for treating iatrogenic ICB. Many drug trials present negative results, and findings showing some efficacy of a specific drug have not been repeated or confirmed in larger study populations. Thus, the evidence level for adjunct pharmacotherapies is generally low, and no drug has been approved for treating iatrogenic ICB.

## 5 Animal Models of Impulsive‐Compulsive Behaviour

Animal models are indispensable for gaining precious insights into the overlapping neural circuitry underlying targets of dopaminergic medications and ICBs. Rodents are the most utilised model due to the extensive etiological and physiological knowledge existing, as well as the considerable genetic and physiological similarities to humans. Consequently, they provide valuable tools for modelling human disease. Substantial effort has been invested into the development of rodent models that recapitulate the features of human ICBs and PD [10], which has helped to establish a strong causal link between dopaminergic medications and the onset of ICBs. Most of the available studies have used behavioural tests developed for rats, although more recently, important findings have been validated in mouse models.

The 6‐hydroxydopamine lesion represents the most common approach used to mimic a PD phenotype by partial dopaminergic denervation in the striatum or in the substantia nigra [36, 37, 38, 39, 40]. Alternatively, the use of adeno‐associated viral vector overexpression of alpha‐synuclein, a pathological hallmark of human PD, has also led to dopamine degeneration, thus creating a model of PD [41, 42]. Reflecting the human PD condition, these lesions can induce deficits in movement initiation and speed (like bradykinesia and akinesia), which are subsequently improved by dopaminergic medications. Although the PD phenotype may confound performance in operant tasks requiring physical responses, the motor deficits are generally mild due to the use of parkinsonian models with mild lesions in all the available studies of ICB in PD. Moreover, several behavioural metrics extrapolated from the tests can be used to dissociate between motor deficits and impulsive phenotypes. In addition, tests based on touch‐screen response systems offer the advantage of facilitating the training procedure in presence of motor abnormalities. Additionally, several studies have reported a development of ICBs also in healthy control animals, emphasising that these behaviours might be primarily driven by drug‐induced dopaminergic dysregulation rather than dependent on the presence of dopaminergic degeneration in PD [10]. This evidence is provided by multiple studies using well‐established behavioural tests, where the D2/D3 agonists ropinirole and pramipexole consistently induce ICBs both in healthy sham controls and animals with dopaminergic depletion modelling PD. Below, we detail the most common behavioural tests used and summarise the main findings linking dopaminergic regimens and the onset of ICBs.

**FIGURE 1:**
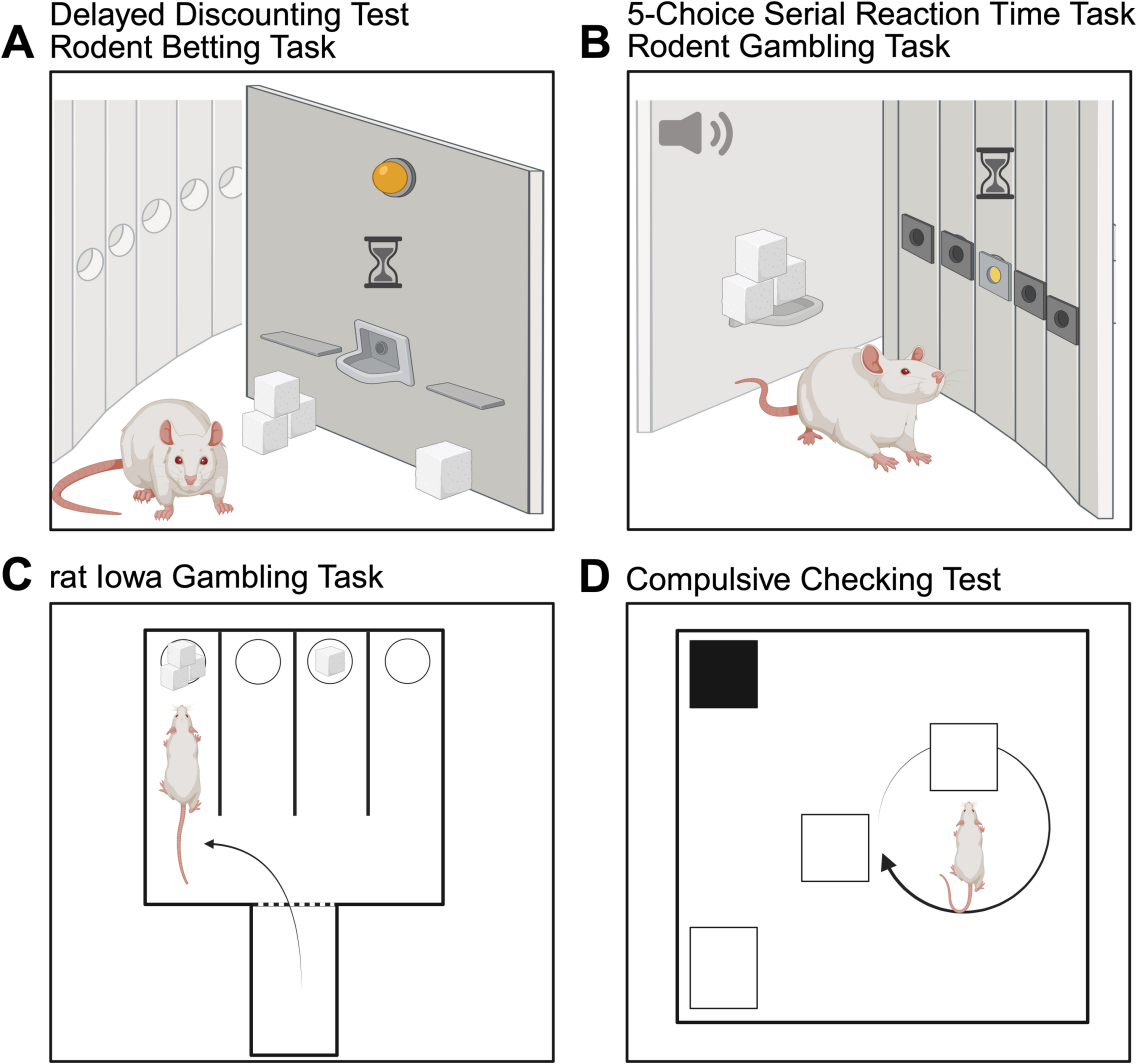
Schematic representation of rodent behavioural tests mimicking human impulsive‐compulsive behaviour. (A) The delayed discounting and the rodent betting task are conducted in operant chambers. They are based on the animal's choice between two options, often presented as levers associated with the delivery of a wager, under conditions of variable reward delays or limited response time. (B) The five‐choice serial reaction time task and the rodent gambling task are also performed in operant chambers. They require the rat to make a nose‐poke response within a specific timeframe in one of the illuminated holes to receive a reward. (C) The manual version of the rat Iowa gambling task is performed in a maze where the rat is free to choose between two arms that are baited with either advantageous or disadvantageous reward choices. (D) The compulsive checking test is performed in an open arena where the rat is free to explore and perform ‘checking’ behaviour directed at different objects. Created in BioRender. Cenci Nilsson, A. (2026, https://BioRender.com/wa0ugsn).

In patients with ICB, a heightened discounting of reward probability and delay can contribute to maladaptive decision‐making, as it favours the pursuit of immediate rewards regardless of their long‐term consequences. These features are preclinically evaluated using test paradigms of probability discounting or delay discounting. Probability discounting tasks assess an animal's tendency to choose small‐but‐certain rewards versus larger‐but‐uncertain ones (greater preference for the large‐uncertain reward indicates greater risk‐seeking/impulsivity). Delay discounting tasks evaluate how much an animal prefers immediate smaller rewards over larger delayed rewards, where a higher preference for immediate rewards indicates greater impulsivity (Figure 1A). The test paradigms to assess delay/probability discounting are essentially the same in rats and mice, although technical aspects related to food reward size, training duration and drug protocols need to be adapted across species to obtain comparable measures of impulsivity. A rat model of probability discounting coupled intracranial self‐stimulation of the lateral hypothalamus with pramipexole administration and found that pramipexole increased probability discounting in both controls and PD‐like rats [38]. Similarly, Holtz et al. [40] found that pramipexole, at doses capable of improving PD‐related motor deficits, increased discounting, and that rats became more impulsive in the test on pramipexole treatment independently of the presence of a PD‐like phenotype.

The 5‐Choice Serial Reaction Time Task (5‐CSRTT) provides high translational validity for studying the cognitive dimensions of attentional deficits and was developed from the continuous performance tests of Rosvald and Mirsky [43] performed in patients with Attentional Deficit/Hyperactivity Disorder. It allows for the precise measurement of sustained attention to a variable number of locations (five different illuminated holes) in order to collect a reward (Figure 1B). Furthermore, the 5‐CSRTT has been successfully translated to the touchscreen platform for rodents [44, 45, 46]. Premature responses reflect a failure of performance on inhibitory controls and are examples of impulsivity, whereas perseverative responses are a form of compulsive maladaptive response [47]. An increased rate of premature responses was reported in animals with PD phenotype induced by alpha‐synuclein overexpression following pramipexole treatment [41]. Another study investigating rats' performance on the inhibition of prepotent responses (stop signal), waiting (delayed gratification) and maintaining the ongoing responses, showed that pramipexole increased both waiting impulsivity and impulsive action. These effects were independent from the PD‐like lesion, though the effect was enhanced in lesioned rats with higher impulsivity traits [42].

The Iowa Gambling Task (IGT) is commonly used to assess decision‐making under uncertainty, measuring an individual's capacity to weigh immediate gains against long‐term consequences. The rodent adaptations of the task (Figure 1B,C) replicate this paradigm by requiring animals to navigate between disadvantageous choices (characterised by large immediate rewards offset by high‐frequency penalties) and advantageous alternatives, which offer smaller individual rewards but yield a higher cumulative return due to infrequent losses [48]. This test has been adapted for rats and mice using maze, operant and touchscreen‐based chambers. Chronic ropinirole alone or combined with levodopa affected the rat's ability to distinguish between a disadvantageous and advantageous choice during the task [37]. Furthermore, chronic ropinirole treatment increased the preference for uncertain options independently of the dopaminergic lesion in the rodent betting task, which mimics a gambling‐like task in rodents (Figure 1A) [39]. An elegant study by Tremblay et al. showed that chronic slow‐release ropinirole treatment increased motor impulsivity in the rodent gambling task (Figure 1B) when reward‐related choices were paired with audio stimuli. The increase in premature response was not associated with an increase in risky decisions, suggesting that ropinirole treatment does not affect all aspects of decision‐making equally [49]. Pramipexole drove a shift towards disadvantageous outcomes in a mouse model of PD during touchscreen IGT sessions. This impairment was specifically rescued by D3 receptor antagonism, highlighting the D3 receptor's critical role in the pathogenesis of impulsive decision‐making [50].

A core component of the ICB spectrum is compulsivity, which is defined by repetitive, nonfunctional actions performed due to uncontrollable internal drives. Repetitive actions occur within the context of punding, where a stereotyped ritualistic manipulation or sorting of objects is driven by an irresistible urge lacking any functional goal [51]. Key aspects of human compulsivity are translated in the rodent compulsive checking test, modelled by repeated administration of the D2/3 agonist quinpirole, which induces excessive repetitive checking of objects within the testing apparatus, and is used as a model of obsessive‐compulsive disorder (Figure 1D) [52]. In a specific genetic strain of mice (A/J background but not C57BL/6 J), chronic quinpirole treatment exacerbated a compulsive phenotype proposed to mirror the ritualistic behaviours seen in obsessive‐compulsive disorders. Interestingly, this contrast between the ritualistic manifestations in A/J mice (as increased behavioural repertoires) and the stereotypies‐prone characteristic of C57BL/6 J (increase behavioural repetitions) under the same testing procedure, highlights the importance of strain selection in modelling obsessive‐compulsive spectrum disorders [53]. Importantly, not only quinpirole, but ropinirole and the combination of ropinirole and levodopa, induced compulsive checking, in control and in rats bearing a PD phenotype [37].

## 6 Pathophysiology and Pharmacological Mechanisms

### 6.1 Human Neuroimaging Studies

Several aspects of ICB have been explored by human imaging studies and reviewed in detail previously, for example, in [11, 54]. The largest body of evidence stems from studies in PD patients, addressing effects of the disease pathology and adverse effects of dopaminergic treatment. Imaging methods enable in vivo visualisation of underlying pathophysiological mechanisms in humans directly, providing insights important for predicting treatment outcomes and possibly guiding future drug development. However, imaging techniques are costly, resulting in smaller patient populations with results harder to reproduce.

Functional Magnetic Resonance Imaging (fMRI) infers neuronal activation by blood oxygen dependent (BOLD) signal. Studies have shown that activation and functional connectivity patterns between key brain areas involved in the reward system and impulse control, such as the ventral striatum, amygdala, the anterior cingulate cortex and prefrontal cortical regions, are altered in PD patients with ICB [11, 55]. Treatment with dopaminergic agents may exacerbate impaired functional connectivity within inhibitory control pathways in PD patients during gambling tasks [56].

Like fMRI, [^15^O]H_2_O Positron Emission Tomography (PET) mirrors metabolic activity of specific brain regions by reflecting regional cerebral blood flow. Treatment with dopaminergic drugs increasing impulsive behaviour has been found to raise regional blood flow to the right medial prefrontal cortex and left posterior cingulate cortex in PD patients [57]. With PET imaging, it is possible to visualise postsynaptic dopamine D2/3 receptor availability using radioligands such as [^11^C]raclopride or [^18^F]fallypride that bind to these receptors, competing with endogenous dopamine. Evidence points towards a higher endogenous dopaminergic state in the ventral striatum as a factor contributing to ICB under dopaminergic therapy [54]. Thus, PD patients with pathological gambling exhibit a higher decrease in [^11^C]raclopride binding potentials in the ventral striatum, suggesting higher dopamine release, during gambling tasks compared to PD patients without ICB [58]. Similarly, Stark et al. [59] demonstrated that PD patients who developed ICB following dopaminergic treatment exhibited lower [^18^F]fallypride binding potentials in the ventral striatum and putamen compared to those without ICB.

**FIGURE 2:**
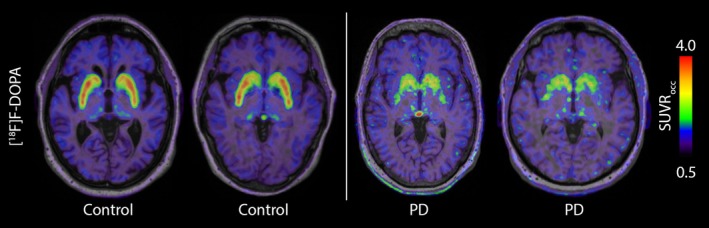
[^18^F]DOPA positron emission tomography (PET) in healthy controls and patients with Parkinson's disease (PD). Images of two healthy controls (left) and two participants with PD (right). Higher [^18^F]DOPA uptake is seen in the striatum of control participants, whereas uptake in PD patients is diminished reflecting the dopamine synaptic loss present in PD. Colour bar denotes standardised uptake value ratios (SUVRs) calculated using the occipital cortex (occ) as reference region where red equals higher [^18^F]DOPA uptake. Participants underwent [^18^F]DOPA PET as part of the Swedish BioFINDER studies (www.biofinder.se).

Further, the dopaminergic system can be studied at a presynaptic level by measuring dopamine transporter availability with PET or Single Photon Emission Computed Tomography (SPECT), reflecting dopaminergic terminal integrity. Most studies show a downregulation of dopamine transporter availability in the ventral striatum of PD patients with ICB [54]. This could be a result of degenerated dopaminergic terminals or related to raised dopamine levels in the synapse, in line with a higher dopamine tone in this region. Accordingly, lower dopamine transporter levels in the ventral striatum have been shown to predispose development of ICB in PD patients [60]. [^18^F]DOPA PET reflects dopaminergic storage in dopaminergic projections to the striatum (Figure 2) but is not as well studied in the field of ICB. One study performed by Hammes et al. [61] using [^18^F]DOPA PET showed that presynaptic dopamine storage capacity in the ventral striatum is negatively correlated with ICB severity in PD patients, pointing towards a degeneration of dopaminergic projections in this region. The only neuropathological study performed in this context found less alpha‐synuclein pathology and a decrease in dopamine D3 receptors in the ventral striatum of PD patients with ICB compared to those without [62]. No difference was found in tyrosine hydroxylase levels, suggesting equal quantity of dopaminergic nerve terminals to the ventral striatum in disagreement with the [^18^F]DOPA PET findings by Hammes et al. [61].

In summary, available evidence suggests a synergistic effect of dopaminergic treatment and predisposing factors, such as an increased dopaminergic tone, in relatively spared non‐motor regions of the striatum as the underlying cause for ICB. A hyperdopaminergic state in these regions is consistent with a downregulation of presynaptic dopamine transporter availability and lower postsynaptic levels of dopamine receptors found in several studies. Possibly, predisposing functional alterations in inhibitory control pathways suggested by fMRI studies mayconcomitantly contribute to ICB development in PD patients and be exacerbated following dopaminergic treatment.

### 6.2 Preclinical Insights Into Neurobiological Mechanisms

Despite the acknowledged limitations of preclinical studies, such as the lack of the slow and heterogeneous pathology of human conditions, as well as the lack of motor and cognitive complexity of humankind, animal models offer the unique advantage of allowing correlation of behavioural changes with underlying neuronal circuitry in brain areas.

Acquired knowledge on neuronal stimulation of the loop connecting the cortex, striatum and thalamus has proven to be essential, confirming its involvement in regulating motor control, motivation and decision‐making, and its dysregulation in ICBs. PET and autoradiography revealed that highly impulsive rats have reduced D2/D3 receptor availability in the nucleus accumbens core compared to low‐impulsive rats [63]. A recent study highlighted that rats exhibiting impulsive behaviours displayed lower levels of the dopamine D2/D3 receptor in the nucleus accumbens, opposite to insular and prelimbic cortices. Selective activation of D3 receptors reduced impulsivity in highly impulsive rats, suggesting a potential association between impulsive choice and D2/D3 receptor expression [64]. Moreover, altered dopamine transporter levels have been linked with the occurrence of impulsive and compulsive behaviours, due to the incorrect balance of dopamine signalling in the striatum and prefrontal cortex. By altering the dopamine transporter availability in the nucleus accumbens using the lentivirus technique, overexpression of the transporter and the consequent reduced dopaminergic tone was related to impulsive choice and risk‐prone phenotype [65].

In summary, animal models coupled with advanced behavioural readouts and neuronal investigations represent a solid foundation for further understanding of the human condition in iatrogenic ICBs. The knowledge gained is crucial in the work to reduce adverse events and to guide the development of treatments for patients based on new therapeutic targets.

## 7 Conclusion and Future Perspectives

Taken together, a large variety of tools and study designs has been developed to study ICB induced by dopaminergic medication during the last decades. In PD, ICB starts to be well‐understood from an epidemiological and clinical perspective, enabling evidence‐based guidelines for ICB management [19]. Gaps in knowledge consist of questions regarding more detailed treatment strategies, such as the effect of adding a dopamine agonist to levodopa therapy or using extended‐release formulations for dopamine agonists. Notably, the body of evidence is substantially smaller in patient populations other than PD patients. Thus, epidemiological and clinical approaches that have been established in PD should be applied to non‐PD populations under dopaminergic therapy in the future. To date, it remains unknown if those populations share clinical characteristics and risk factors with PD patients. Considering the large number of RLS patients at risk for iatrogenic ICB, RLS patients should receive the main attention when transferring methods from PD populations. In addition, treatment options other than dopaminergic agents should be the first choice in RLS patients at risk for ICB. The gabapentinoids gabapentin and pregabalin were developed as anticonvulsants and have gained importance in RLS treatment [66]. Although known adverse events of gabapentinoids include dizziness and fatigue, they have not been reported to increase ICB.

Interventional trials in patients have struggled to identify efficacious pharmacological treatment for ICB, possibly due to limited sample size in relation to the large individual response variability in ICB studies. At the same time, neuroimaging studies have started to reveal possible pathophysiological mechanisms of iatrogenic ICB, supported by animal models resembling human ICB under dopaminergic therapy. Broadening the exploration of therapeutic targets and candidate drugs in animal models of ICB guided by human neuroimaging studies seems like a feasible and cost‐effective strategy for the future. Understanding the brain connectivity and signalling pathways involved in iatrogenic ICB would on the one hand enable the development of safer dopaminergic drugs with less ICB as adverse event. On the other hand, identifying an agent that can reduce impulsivity and compulsivity in general could be a game changer for many patient groups, given that these maladaptive behavioural features constitute a problematic aspect of many psychiatric disorders beyond ICBs.

## Funding

This work was supported by Research Council of Svenska Spel AB (FO2022‐0008).

## Conflicts of Interest

The authors declare no conflicts of interest. M.W. has obtained funding from the research council of Svenska Spel AB (Grant Number FO2022‐0008). J.T. has received compensation for speaking engagements and consultancies from AbbVie and TransPerfect. R.S. has received consultancy/speaker fees from Eli Lilly, Novo Nordisk, Roche and Triolab. P.O. has received consultancy/speaker fees from AbbVie, Bial, Britannia, Convatec, Merz, Nordic Infucare and Zambon. M.A.C.'s and E.E.'s work in this area is supported by project grants from the Swedish Research Council (Grants 2020‐02696 and 2024‐02491), the Lundbeck Foundation (Grant R336‐2020‐1035) and the Swedish Government Funding for Clinical Research (Grant ALF‐projekt 43301). A.H. has a position at Lund University that is sponsored by the state‐owned gambling operator AB Svenska Spel and has funding acquired from the research council of Svenska Spel. We thank the strategic research area MultiPark for infrastructure support.

## Data Availability

Data sharing not applicable to this article as no datasets were generated or analysed during the current study.
